# Neural Network Approach to Investigating the Importance of Test Items for Predicting Physical Activity in Chronic Obstructive Pulmonary Disease

**DOI:** 10.3390/jcm12134297

**Published:** 2023-06-27

**Authors:** Yoshiki Nakahara, Shingo Mabu, Tsunahiko Hirano, Yoriyuki Murata, Keiko Doi, Ayumi Fukatsu-Chikumoto, Kazuto Matsunaga

**Affiliations:** 1 Graduate School of Sciences and Technology for Innovation, Yamaguchi University, Yamaguchi 7558611, Japan; 2 Department of Respiratory Medicine and Infectious Disease, Yamaguchi University Hospital, Yamaguchi 7558505, Japan

**Keywords:** COPD, physical activity, prediction, neural network, autoencoder

## Abstract

Contracting COPD reduces a patient’s physical activity and restricts everyday activities (physical activity disorder). However, the fundamental cause of physical activity disorder has not been found. In addition, costly and specialized equipment is required to accurately examine the disorder; hence, it is not regularly assessed in normal clinical practice. In this study, we constructed a machine learning model to predict physical activity using test items collected during the normal care of COPD patients. In detail, we first applied three types of data preprocessing methods (zero-padding, multiple imputation by chained equations (MICE), and k-nearest neighbor (kNN)) to complement missing values in the dataset. Then, we constructed several types of neural networks to predict physical activity. Finally, permutation importance was calculated to identify the importance of the test items for prediction. Multifactorial analysis using machine learning, including blood, lung function, walking, and chest imaging tests, was the unique point of this research. From the experimental results, it was found that the missing value processing using MICE contributed to the best prediction accuracy (73.00%) compared to that using zero-padding (68.44%) or kNN (71.52%), and showed better accuracy than XGBoost (66.12%) with a significant difference (*p* < 0.05). For patients with severe physical activity reduction (total exercise < 1.5), a high sensitivity (89.36%) was obtained. The permutation importance showed that “sex, the number of cigarettes, age, and the whole body phase angle (nutritional status)” were the most important items for this prediction. Furthermore, we found that a smaller number of test items could be used in ordinary clinical practice for the screening of physical activity disorder.

## 1. Introduction

Chronic obstructive pulmonary disease (COPD) is a chronic inflammatory disease of the lungs that is primarily caused by smoking, which causes the occlusion of airflow and prevents the return to normal function according to respiration function tests [1]. Airflow limitation and air trapping lead to the static and dynamic hyperinflation of the lungs. In turn, this causes patients to have difficulty breathing during laborious tasks, leading to a reduction in exercise tolerance and physical activity as measured by the indicators of activity time and the amount of physical activity at each level of exercise intensity. This is called physical activity disorder. In other words, if COPD patients suffer from reductions in their exercise ability, this leads to reduced physical activity, which in turn leads to a vicious cycle of reduced muscle strength and worse prognosis [2,3,4,5]. In addition, COPD patients may not be aware of their respiratory symptoms because they are physically inactive, i.e., some patients are physically inactive even though they feel few respiratory symptoms. This suggests that it is difficult to make immediate judgements regarding actual physical activity in clinical settings. Physical activity is regulated by various factors, which can make physical activity disorder challenging to understand and assess in patients with COPD. However, the mechanism of physical activity disorder that worsens COPD and appropriate treatment targets have not been found. In addition, there are many related test items and running all possible tests incurs significant costs. As a result, in the treatment of COPD, it is important to find relevant test items to predict physical activity with high accuracy.

Previous research on predicting physical activity has included predictions made with and without machine learning. As an example of predictions using machine learning, pulse rate data were used to predict the physical activity of elderly patients [6]. In [7], the physical activity of children not yet in preschool was predicted using a random forest method. In [8], data provided by sensor chips capable of being adapted for use in mobility assistance devices, such as canes or crutches, were used to make predictions using machine learning. In [9], accelerometer bracelets were used to predict physical activity using various machine learning models. In [10], physical activity was predicted using experimental processes of change (experimental POCs), behavioral processes of change (behavioral POCs), and interactions between the two. Research using deep learning has also been conducted, including a proposed physical activity prediction model that used long short-term memory recurrent neural networks [11]. In [12], factors associated with low-intensity physical activity were examined using multivariate regression analysis. In [13], the effectiveness of a simple prediction equation for step counts obtained using multivariate regression analysis was evaluated by comparing the predicted values to actual measured values. Some research has been conducted without using machine learning, including [14], which investigated whether it was possible to predict domain-specific physical activity 20 years later in adulthood using domain-specific physical activity in early childhood. Similarly, Ref. [15] investigated the extent to which adult physical activity patterns could be predicted using physical characteristics, physical abilities, and activity levels during adolescence. In [16], physical activity and metabolic equivalents (METs) per day were predicted using three commercially available accelerometers and eight regression equations. Ref. [17] applied the Fogg behavior model and the transtheoretical behavior model to predict physical activity. In [18], the authors examined the association between physical activity and the expiratory to inspiratory (E/I) ratio of mean lung density (MLD) and showed that the E/I ratio of MLD could be a useful imaging biomarker for the early detection of physical inactivity in COPD patients.

On the other hand, machine learning models for predicting the physical activity of COPD patients using multifactorial test data, including blood, lung function, walking, and chest imaging tests, have not been constructed. Therefore, in this study, we attempted to build a typical neural network model for physical activity prediction using various types of test items and evaluated its prediction performance. Generally, in machine learning, a large amount of training data is required. However, since collecting a sufficient amount of data for specific diseases from one medical institution is difficult, the proposed prediction model applied pre-training using an autoencoder [19]. Since physical activity is defined by a variety of factors, it is difficult to predict physical activity disorder using simple tests in ordinary clinical practice. Therefore, the main aims of this study were as follows: (1) to clarify whether physical activity could be predicted using machine learning and measure the prediction performance; (2) to clarify which test items were important for prediction using a method called permutation importance; (3) to examine the applicability of machine learning for the diagnosis of physical activity disorder through (1) and (2), even in local hospitals with access to only simple tests.

## 2. Materials and Methods

### 2.1. Ethics Approval and Consent to Participate

This study was approved by the Institutional Review Board of Yamaguchi University Hospital (H27-204) and was conducted according to the principles of the Declaration of Helsinki. Informed consent was obtained from all individuals included in this study.

### 2.2. Dataset

The dataset used in this study was composed of 406 cases of patient data provided by the Department of Respiratory Medicine and Infectious Disease, Yamaguchi University Hospital, Japan (COPD patients, non-COPD patients, and healthy individuals). The non-COPD patients were patients with respiratory illnesses other than COPD, such as bronchial asthma, bronchiectasis, and interstitial pneumonia. The numbers of patients in each category were 143 COPD patients, 238 non-COPD patients, and 25 healthy individuals. Data were collected for 32 test items, including questionnaires regarding respiratory symptoms, whole body phase angle, blood tests, lung function tests, and computed tomography (CT) images. The activity monitor we used (produced by Omron) measured and aggregated exercise (Ex) from the number of steps and activity levels during each hour from 0:00 to 23:00. Ex was computed by multiplying activity intensity (METs) by time, and only exercise with an intensity of 3.0 METs or more was used to calculate Ex. Physical activity represents the performance of physical actions, so smaller values indicated reductions in physical activity. Total exercise (T-Ex) values of less than 3 (METs × hr) were defined as abnormal physical activity, while those of 3 (METs × hr) or more were considered normal physical activity, as previously reported in [20]. Based on the above data, we trained a neural network to classify each patient as displaying either normal or abnormal physical activity. The explanatory variables were the 32 test items (the inputs to the neural network) and the response variable was physical activity (i.e., Total Exercise or T-Ex (the output from the neural network)). When applying these data to machine learning, each value was normalized to fall between a minimum of 0 and a maximum of 1. Detailed information about the variables is presented in Table 1. Note that importance index in Table 1 will be explained in later sections. However, as not every test was performed for each patient, the data were missing some values. Thus, one of the three missing value imputation methods (zero-padding, multiple imputation by chained equations (MICE) [21], and k-nearest neighbor (kNN) [22]) was used to complement missing values.

### 2.3. Missing Value Processing

The outlines of the three missing value processing methods (zero-padding, MICE, and kNN) are as follows:

- Zero-padding just replaces missing values with zeros;
- MICE handles missing values by predicting them (in detail, MICE builds a machine learning model that predicts missing values for certain test items by using the values of the other test items);
- kNN handles missing values by using values of the nearest data from the data with missing values.

The detailed procedures for MICE and kNN are as follows:

- MICE is a multiple imputation method in which missing values are predicted through regression using other feature values. Below, the explanatory variables of the dataset (the values of each of the test items) are referred to as $x_{1},x_{2},\cdots ,x_{q}$ in the MICE sequence. The explanatory variables are called feature values hereinafter.
  1. An initial value (e.g., mean value) is assigned to each missing value to create pseudo-complete data;
  2. If $x_{1}$ is a missing value, $x_{1}$ is returned to the missing state and a regression model (e.g., linear regression) is used on the pseudo-complete data $x_{2},x_{3},\cdots ,x_{q}$ to produce and replace $x_{1}$ with the imputed value;
  3. The imputed values for $x_{2}$ and onward are generated and replaced, just as in step 2;
  4. The procedure from steps 2–3 is repeated an arbitrary number of times. MICE is a method that updates imputed values sequentially. In this study, the initial values were set as the means of the corresponding features and linear regression was used.
- kNN is a supervised learning classification algorithm. In kNN, the given data are plotted on a feature space and if unknown class data appear, the *k* points of the data are selected in the order of proximity to the unknown class data and the unknown class data are categorized into classes based on the majority of these *k* points. Euclidean distance was used to compute distance and k=1, i.e., the k-nearest neighbor algorithm, was used for missing value processing in our preliminary experiments. In kNN with k=1, the nearest data to the missing values are searched and the values of the nearest data are used to complement the missing values. If the nearest data also have missing values, the next nearest data without missing values are chosen instead.

### 2.4. Physical Activity Prediction Model Using an Autoencoder

In this section, we first summarize how we constructed a neural network for physical activity. There were two steps for the construction. The type of neural network trained in the first step was an “autoencoder”. The autoencoder was constructed so as to extract important information from the inputs (test items). This was called “pre-training”. Then, in the second step, a simple “feed-forward neural network” was constructed by changing the neural network structure built in step 1 to predict physical activity levels. This was called “fine-tuning”.

Then, 3- and 4-layer neural networks (NNs) were used to build prediction models and each model was constructed using a combination of pre-training with the autoencoder and fine-tuning. The autoencoder was composed of an encoder, which converted the inputs into feature values, and a decoder, which reconstructed the inputs based on the obtained feature values. The goal of the pre-training was to set the initial weights of the NNs for the training that made the inputs and outputs coincide. The pre-training had the effect of constructing an effective encoder that extracted features that were hidden in the test items. Following pre-training, fine-tuning was conducted to achieve the final objective, i.e., the prediction of T-Ex.

**Figure 1:**
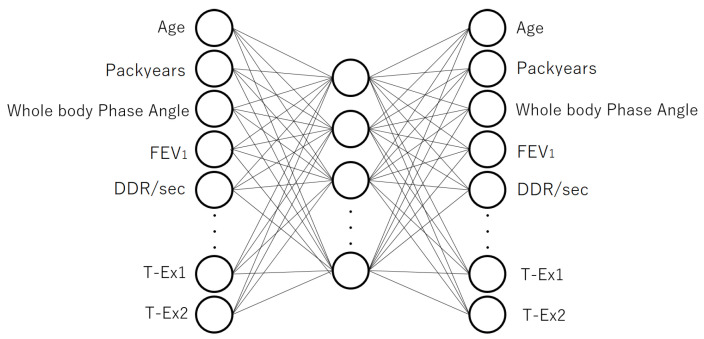
The model structure of the 3-layer autoencoder used for pre-training. There were 34 units in the input layer. In the hidden layer, a feature vector (feature values) that encoded the inputs was obtained. There were 34 units in the output layer, in which the inputs were reconstructed from the hidden layer vectors. The number of units in the hidden layer was 20, i.e., the feature values were represented by 20-dimensional vectors.

**Figure 2:**
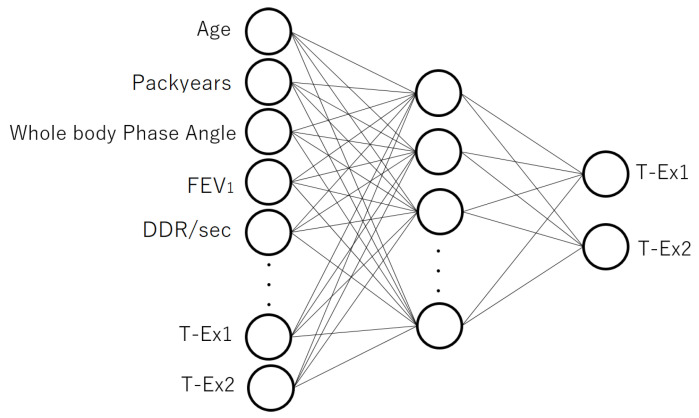
The model structure used for the fine-tuning, which was constructed after pre-training with the 3-layer autoencoder. The output layer in Figure 1 was removed and the model was connected to a new output layer to predict T-Ex1 and T-Ex2.

Figure 1 shows the 3-layer autoencoder that conducted the pre-training. Figure 2 shows the model structure used for the fine-tuning, which was constructed after pre-training. In Figure 1, the number of input units was 34, where 32 units corresponded to the test items (except T-Ex) and two units corresponded to T-Ex1 and T-Ex2. The number of output units was also 34, corresponding to the number of input units. There were two T-Ex units to represent the one-hot vectors for normal and abnormal activity, i.e., the normal case was represented as (T-Ex1, T-Ex2) = (1,0), while the abnormal case was represented as (T-Ex1, T-Ex2) = (0,1). The loss function used in the autoencoder training was the mean squared error (MSE).

Next, a classifier was constructed using the autoencoder (Figure 2) to conduct fine-tuning. As the goal was to predict T-Ex, T-Ex could not be used as an input at this stage. Accordingly, when conducting fine-tuning, zeros were used as the inputs for the units corresponding to T-Ex. The two units in the output layer produced the respective probability that the input was normal or abnormal. The loss function used for fine-tuning was cross-entropy. Note that the weights of the input and hidden layers were adjusted in pre-training and further updated in fine-tuning.

**Figure 3:**
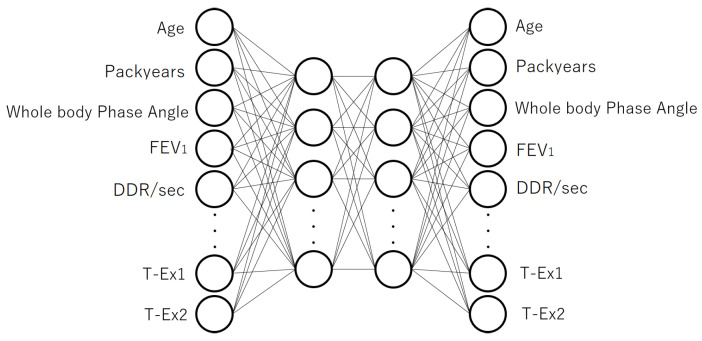
The model structure of the 4-layer autoencoder used for pre-training. The input layer had 34 units. In the two hidden layers, the vectors (feature values) that encoded the inputs were obtained. The output layer had 34 units and the inputs were reconstructed from the hidden layer vectors.

**Figure 4:**
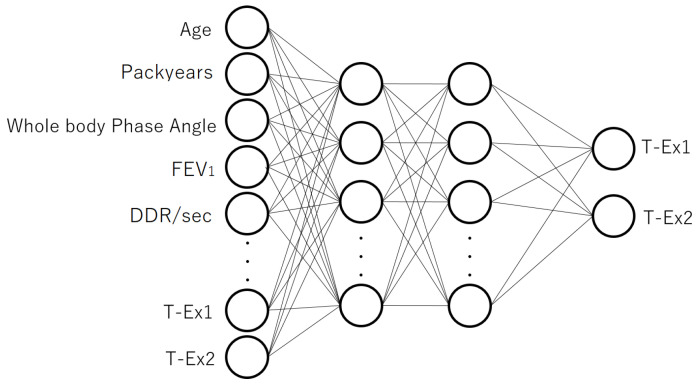
The model structure used for fine-tuning, which was constructed after pre-training with the 4-layer autoencoder. The output layer in Figure 3 was removed and the model was connected to a new output layer to predict T-Ex1 and T-Ex2.

For comparison, we also constructed a model based on a 4-layer autoencoder. The 4-layer autoencoder and the structure for fine-tuning are shown in Figure 3 and Figure 4, respectively. The numbers of input and output units were the same as in the 3-layer case, while there were 20 units in both the first and second hidden layers. Representation ability increases with the number of neural network layers; however, because the amount of treated data was limited in this study, it was possible that overfitting could occur simply by deepening the number of layers. To prevent this overfitting, dropout [23] was applied to the first and second layers of the autoencoder (the dropout rate was 20% for the first layer and 10% for the second layer).

### 2.5. Calculating the Importance of Each Test Item

Permutation importance [3] was used to calculate the importance of each test item. Permutation importance is a method of randomly substituting feature values between data. Using this process, the substituted feature values stop correlating with the response variable, that is, they lose their ability to explain the response variable. By comparing the accuracy when using the substituted data ($acc_{substitute}$) and the accuracy prior to substitution ($acc_{original}$), we could calculate to what extent the feature values contributed to the prediction. The importance of a test item was calculated using Equation (1).

(1) $$importance\left(i\right)=acc_{original}\left(i\right)-acc_{substitute}\left(i\right),$$

where *i* is a test item with values that have been substituted to calculate its importance.

## 3. Evaluation Metrics and Results

### 3.1. Evaluation Metrics

In this section, we explain the method for separating the dataset into training and testing datasets. Here, the word “testing” is used to represent the testing dataset, while “test” is used for medical tests. The data used in this study included test results for the same patients on different days. Separating the dataset without taking this into account produced the possibility that test results from the same patient were included in both the training and testing datasets. As reliable prediction results could not be obtained in such cases, we ensured that data from the same patient were not included in either the training or testing datasets. Performance was then assessed using 20-fold cross-validation.

### 3.2. Prediction Accuracy of Each Method

Pairing datasets processed using one of the three missing value imputation methods with one of the four types of neural networks (NNs) (i.e., 3- and 4-layer NNs without pre-training and those with pre-training) yielded a total of 12 condition patterns. The mean accuracy for each method is shown in Table 2. The 3-layer NN that was pre-trained with missing values processed using MICE (3-layer NN with PT (MICE)) had the highest accuracy of 73.00%. In addition, focusing on missing value processing, MICE yielded the highest prediction accuracy compared to zero-padding (*p* = 0.00025) and kNN (*p* = 0.023). Conventionally, XGBoost [24] is often used in the analysis of table data; thus, a comparison between XGBoost, MICE, and the proposed method was also conducted. The accuracy of the proposed method was 73.00%, which was higher than that of XGBoost (66.12%) with a significant difference (p=0.016). Therefore, the 3-layer NN with PT (MICE), i.e., the method with the highest accuracy, was used for the subsequent experiments.

### 3.3. Importance of Each Test Item

**Figure 5:**
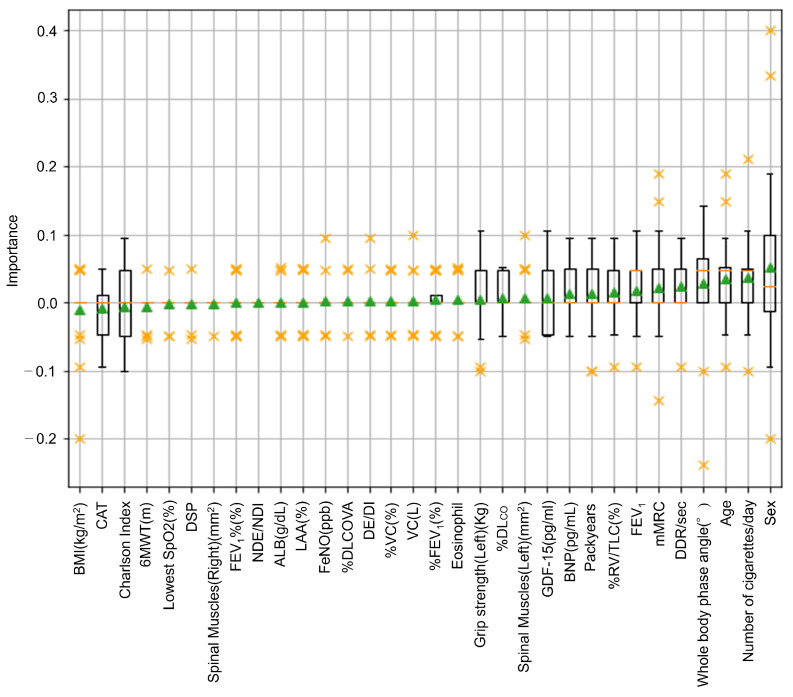
The importance of each test item. The horizontal axis shows the test items and the vertical axis shows their importance. The test items in order of effect on prediction performance were sex, the number of cigarettes per day, age, whole body phase angle, DDR, mMRC, FEV_1_, %RV/TLC, pack years, and BNP.

We calculated the permutation importance of each test item. In Figure 5, the importance of each test item is shown in a box plot. The triangles show the mean values and the test items are sorted by their mean values. Table 1 shows detailed information about the data statistics and their importance. In Table 1, the items are listed in order of importance. As previously stated, larger importance values indicated more important test items. In Figure 5, it can be seen that sex, the number of cigarettes per day, age, whole body phase angle, DDR, mMRC, FEV_1_, %RV/TLC, pack years, and BNP were the most important test items (in this order). Interestingly, whole body phase angle (nutrition status) was the fourth most important test item. Test items showing an importance of 0 or less did not contribute to the prediction or could have been noise due to the effects of individual differences. The lifestyles of the patients also influenced their physical activity, so large deviations in importance were also found for some test items.

### 3.4. Comparison of Classification Performance by the Number of Items Used

Making it possible to predict physical activity not only in large hospitals but also in regional clinics could contribute to the treatment of COPD by reducing test costs. Accordingly, to ascertain whether it would be possible to perform predictions using a smaller number of test items, we also inspected how classification performance changed as the number of test items decreased. Below are the five conditions that were used for this investigation.

1. 32 items (all of the test items);
2. 28 items (removing blood test);
3. 19 items (removing blood test and lung function test);
4. 15 items (removing blood test, lung function test, and walking test);
5. 10 items (removing blood test, lung function test, walking test, and chest imaging test).

Condition 1 was the case in which all 32 test items were used, while conditions 2 to 5 removed the blood test, lung function test, walking test, and chest imaging test one by one. Note that the items removed from conditions 2 to 5 were determined by referencing the items with the lowest importance in Figure 5 and considering the costs of the tests.

Table 3 shows the accuracy, precision, sensitivity, and specificity of each condition. As expected, the highest accuracy, precision, sensitivity, and specificity were obtained when all 32 items were used. However, even in condition 5, an accuracy of 69.28% was obtained, that is, the difference between conditions 1 and 5 was 3.72%. The test items used in condition 5 were age, sex, BMI, whole body phase angle, grip strength (left hand), Charlson index, pack years, the number of cigarettes per day, CAT, and mMRC. The p-values between the accuracy of condition 1 and the accuracy of the other conditions did not show any significant differences; therefore, a smaller number of test items could be conducted in ordinary clinical practice for the screening of physical activity disorder.

### 3.5. Sensitivity of T-Ex by Range

In this study, all T-Ex values of less than 3 were classified as abnormal, but a T-Ex value of less than 1.5 is defined as physical inactivity and implies a poor prognosis [25]. As a result, for patients with T-Ex values of 1.5 or less, there is a particularly prioritized need for physicians to respond, so it would be preferable to be able to predict these values without overlooking any cases. Accordingly, in this section, we analyze the sensitivity of T-Ex by range (Table 4). The analysis was performed using a prediction model for all 32 items. In this investigation, cases were divided into 2.5≤T-Ex<3.0, 2.0≤T-Ex<2.5, 1.5≤T-Ex<2.0, and T-Ex<1.5 and the sensitivity was calculated for each range. As can be seen from Table 4, the number of cases in which 2.5≤T-Ex<3.0 was 217, of which 166 cases were correctly predicted to be abnormal with a sensitivity of 76.50%. The sensitivity for 2.0≤T-Ex<2.5 was 79.46%, while that for 1.5≤T-Ex<2.0 was 85.62% and that for T-Ex<1.5 (severe physical activity reduction) was 89.36%. The sensitivity increased for patients with lower T-Ex values, indicating that the trained model captured the features of physically inactive patients.

### 3.6. Classification Performance for Healthy Individuals, Non-COPD Patients, and COPD Patients

We also analyzed the classification performance for healthy individuals, non-COPD patients, and COPD patients (Table 5). To obtain these results, the same trained model was used for all three patient categories. Note that the data to be predicted did not provide the model with any information regarding which category the patients belonged to.

As shown in Table 5, extreme values for sensitivity (0%) and specificity (100%) were found for healthy individuals. Table 6 shows a confusion matrix of the classification results for healthy individuals, for which only three positive cases were found in the dataset. Accordingly, it is possible that training was insufficient due to the lack of positive cases among the healthy individuals. It is also possible that the test results obtained for healthy individuals rarely showed any signs of physical activity reduction.

The predictions for COPD and non-COPD patients, as shown in Table 5, had high sensitivity and low specificity, which was contrary to the results for healthy individuals. Therefore, the features of positive cases were captured well. In the test results for COPD patients and non-COPD patients, the characteristics of positive cases were strongly expressed, while it was more difficult to make negative classifications. Table 7 and Table 8 show the confusion matrices of the classification results for COPD patients and non-COPD patients, respectively. For patients with COPD, out of the 94 actual positive cases, 89 cases were correctly classified as positive. For non-COPD patients, out of the 120 actual positive cases, 94 cases were correctly classified as positive, indicating a weaker expression of features for reduced physical activity in comparison to that for COPD patients.

## 4. Discussion

In this study, test results from patients with respiratory illnesses, including COPD, who were examined at the Yamaguchi University Hospital in Japan were used to construct a physical activity prediction model. An analysis was performed to determine which test items were important for predicting physical activity. While this study shared the same goal as previous studies in terms of the prediction of physical activity, we aimed to use a different motivation and technical approach. First, if physical activity could be predicted using simple tests that could be conducted in daily clinical practice, the efficiency of COPD treatment could be improved, even in areas with limited medical resources. Second, we implemented two measures to increase the applicability of machine learning to insufficient datasets. The first measure was the enhancement of the amount of data using missing value imputation. As it is difficult to conduct all possible tests on all patients, many missing values were included in the dataset. Accordingly, the following three missing value imputation methods were applied to enhance the data: zero-padding, MICE, and kNN. The second measure was the enhancement of feature extraction through the pre-training of the neural network using an autoencoder. By extracting useful information from test items measured during ordinary clinical practice, we strove to improve the performance of our prediction model for physical activity using machine learning. The results of our experiments indicated that the neural network that was pre-trained using the autoencoder with missing value imputation performed using MICE was the most effective model. Sex, the number of cigarettes per day, age, and whole body phase angle were the most important test items. This implied that it could be possible to conduct screening for reduced physical activity using some low-cost test items while omitting other tests, such as pulmonary function tests. Moreover, the fact that whole body phase angle was a useful item allowed us to infer that poor nutritional status could be related to poor prognosis accompanying physical inactivity. This suggests that in the future, there could be a need to longitudinally examine whether nutrition treatment interventions are effective as new methods of treatment for physical activity disorder. Although there have been studies attempting to find relationships between COPD patients and physical activity/muscle strength/body composition using whole body phase angle [26,27,28], to date, multifactorial analysis using machine learning, including blood, lung function, walking, and chest imaging tests, has not been performed. Therefore, this study showed that whole body phase angle was an important indicator for predicting physical activity.

This study had several limitations. First, there is room for improvement in the accuracy of 73.00%. As the amount of physical activity among patients is related to their personality and environment, it is difficult to obtain accurate predictions based solely on clinical test results. Further improvements in prediction performance may be aided by the collection of new data obtained via questionnaires concerning the living environments of patients. However, the prediction sensitivity for T-Ex < 1.5 was 89.36%, a level that could be clinically helpful as a screening method for reduction in physical activity. Furthermore, when calculating the importance of the test items, we only focused on single items to determine how much they contributed to the classification. If several highly correlated feature values existed, it could be possible that some truly important items were regarded as less important as the information could be imputed from other items, even with the removal of important items. Accordingly, it may be possible to extract the truly important items by calculating item importance after simultaneously shuffling highly correlated items. From another perspective, by changing combinations when shuffling multiple items, it may be possible to discover that although certain items have a small effect on classification, they could have a large effect when combined with other items. Additionally, only one type of data from Yamaguchi University Hospital was used in this study. However, the proposed model is universally applicable to table-form datasets, including those with missing values; thus, we will apply it to other datasets and evaluate its performance.

## Figures and Tables

**Table 1:** The basic data statistics for each test item and the statistics of their permutation importance. The test items are sorted by their mean importance. The data statistics for Sex (M/F) show the numbers of males and females. Note: CI, confidence interval; STD, standard deviation; DDR, desaturation distance ratio; mMRC, modified British medical research council; FEV_1_, predicted forced expiratory volume in 1s (%); RV, residual volume; TLC, total lung capacity; BNP, brain natriuretic peptide; GDF-15, growth differentiation factor-15; DLCO, diffusing capacity of the lungs for carbon monoxide; VC, vital capacity; VA, alveolar volume; DE/DI, the ratio of mean lung density at the end of expiration (E) to the end of inspiration (I); FeNO, fractional exhaled nitric oxide; LAA, low-attenuation area; ALB, albumin; NDE/NDI, the ratio of normal density at the end of expiration (E) to the end of inspiration (I); DSP, distance–saturation product; CAT, COPD assessment test; BMI, body mass index.

	Data Statistics		Importance Index						
	Mean	STD	Mean	Min	1st Quartile	Median	3rd Quartile	Max	95% CI (Lower–Upper)
Sex (M/F)	309/97		0.051	−0.200	−0.013	0.024	0.100	0.400	−0.013–0.116
# of Cigarettes/Day	16.28	14.10	0.038	−0.100	0.000	0.048	0.051	0.211	0.008–0.068
Age	70.15	9.57	0.035	−0.095	0.000	0.049	0.053	0.190	0.003–0.066
Whole Body Phase Angle (${}^{\circ }$)	5.07	0.76	0.028	−0.238	0.000	0.048	0.064	0.143	−0.011–0.066
DDR/s	5.91	6.09	0.024	−0.095	0.000	0.000	0.050	0.095	0.003–0.046
mMRC	0.68	0.89	0.023	−0.143	0.000	0.000	0.051	0.190	−0.012–0.058
FEV_1_	2.19	0.64	0.017	−0.095	0.000	0.048	0.048	0.105	−0.005–0.040
%RV/TLC (%)	95.52	16.81	0.015	−0.095	0.000	0.000	0.048	0.095	−0.005–0.035
Pack Years	28.17	20.67	0.014	−0.100	0.000	0.000	0.050	0.095	−0.011–0.039
BNP (pg/mL)	38.08	49.48	0.013	−0.050	0.000	0.000	0.050	0.095	−0.007–0.032
GDF-15 (pg/mL)	1245	853	0.008	−0.050	−0.048	0.000	0.048	0.105	−0.017–0.032
Spinal Muscles (Left) (mm^2^)	1550	436	0.007	−0.053	0.000	0.000	0.000	0.100	−0.008–0.023
%DLCO	96.25	27.25	0.007	−0.050	0.000	0.000	0.048	0.053	−0.008–0.023
Grip Strength (Left) (Kg)	29.43	8.51	0.005	−0.100	0.000	0.000	0.048	0.105	−0.018–0.028
Eosinophil	3.63	4.60	0.005	−0.050	0.000	0.000	0.000	0.053	−0.008–0.018
%FEV_1_ (%)	85.28	21.28	0.005	−0.050	0.000	0.000	0.012	0.050	−0.010–0.019
VC (L)	3.33	0.81	0.003	−0.050	0.000	0.000	0.000	0.100	−0.013–0.018
%DLCOVA (%)	88.32	38.08	0.002	−0.050	0.000	0.000	0.000	0.050	−0.007–0.011
DE/DI	0.96	0.03	0.002	−0.050	0.000	0.000	0.000	0.095	−0.011–0.016
%VC (%)	99.95	17.04	0.002	−0.050	0.000	0.000	0.000	0.050	−0.009–0.014
FeNO (ppb)	33.70	31.40	0.002	−0.050	0.000	0.000	0.000	0.095	−0.011–0.016
LAA (%)	0.23	0.04	0.000	−0.050	0.000	0.000	0.000	0.050	−0.015–0.015
ALB (g/dL)	4.20	0.31	0.000	−0.050	0.000	0.000	0.000	0.053	−0.013–0.013
NDE/NDI	0.92	0.03	0.000	0.000	0.000	0.000	0.000	0.000	—
FEV_1_% (%)	66.62	12.29	−0.000	−0.050	0.000	0.000	0.000	0.050	−0.015–0.015
Spinal Muscles (Right) (mm^2^)	1586	437	−0.003	−0.050	0.000	0.000	0.000	0.000	−0.008–0.003
DSP	38,082	8404	−0.003	−0.053	0.000	0.000	0.000	0.050	−0.012–0.007
Lowest SpO2 (%)	91.98	3.83	−0.003	−0.050	0.000	0.000	0.000	0.048	−0.012–0.006
6MWT (m)	412.40	85.02	−0.005	−0.053	0.000	0.000	0.000	0.050	−0.015–0.005
Charlson Index	0.96	1.04	−0.006	−0.100	−0.048	0.000	0.048	0.095	−0.032–0.021
CAT	10.87	7.44	−0.007	−0.095	−0.048	0.000	0.012	0.050	−0.027–0.012
BMI (kg/m^2^)	22.73	3.60	−0.010	−0.200	0.000	0.000	0.000	0.050	−0.037–0.017

**Table 2:** The mean accuracy obtained by each method. The 3-layer NN with PT (MICE) showed the highest accuracy.

Method	Accuracy (%)
3-layer NN without PT (zero-padding)	68.44
3-layer NN without PT (MICE)	72.55
3-layer NN without PT (kNN)	70.32
4-layer NN without PT (zero-padding)	65.98
4-layer NN without PT (MICE)	72.66
4-layer NN without PT (kNN)	69.74
3-layer NN with PT (zero-padding)	68.05
3-layer NN with PT (MICE)	73.00
3-layer NN with PT (kNN)	71.52
4-layer NN with PT (zero-padding)	67.63
4-layer NN with PT (MICE)	72.81
4-layer NN with PT (KNN)	70.05

Note: PT, pre-training.

**Table 3:** The classification performance using different numbers of test items. The classifier using all 32 items showed the best accuracy, precision, sensitivity, and specificity. On the other hand, even when the number of test items was reduced to 10, the accuracy was still approximately 69%. The *p*-values show the results of t-tests on accuracy between condition 1 and the other conditions and demonstrate that there were no significant differences.

# of Items	Accuracy	Precision	Sensitivity	Specificity	*p*-Value (vs. Condition 1)
32	73.00	75.34	76.59	69.40	–
28	70.78	73.07	73.49	67.79	0.178
19	69.42	70.23	72.64	69.10	0.106
15	69.05	72.09	73.66	67.65	0.055
10	69.28	71.09	73.66	67.08	0.182

**Table 4:** The sensitivity by T-Ex range. TP (true positive) represents the number of correctly predicted positive cases as positive and FN (false negative) represents the number of incorrectly predicted positive cases as negative. Therefore, TP + FN represents the actual positive numbers. Sensitivity was calculated using TP/(TP + FN).

	2.5 ≤ T-Ex < 3.0	2.0 ≤ T-Ex < 2.5	1.5 ≤ T-Ex < 2.0	T-Ex < 1.5
TP	166	147	125	84
TP + FN	217	185	146	94
Sensitivity (%)	76.50	79.46	85.62	89.36

**Table 5:** The classification performance for healthy individuals, non-COPD patients, and COPD patients.

	Accuracy (%)	Precision (%)	Sensitivity (%)	Specificity (%)
Healthy Individuals	88.00	0.00	0.00	100.0
Non-COPD Patients	68.49	70.27	65.00	72.03
COPD Patients	78.32	77.88	93.62	48.98

**Table 6:** The confusion matrix of the classification results for healthy individuals.

		Prediction	
		Positive	Negative
Actual	Positive	0	3
	Negative	0	22

**Table 7:** The confusion matrix of the classification results for COPD patients.

		Prediction	
		Positive	Negative
Actual	Positive	88	6
	Negative	25	24

**Table 8:** The confusion matrix of the classification results for non-COPD patients.

		Prediction	
		Positive	Negative
Actual	Positive	78	42
	Negative	33	85

## Data Availability

The dataset used in this study is not publicly available due to ethical constraints.
